# Impact of central and peripheral estrogen treatment on anxiety and depression phenotypes in a mouse model of postmenopausal obesity

**DOI:** 10.1371/journal.pone.0209859

**Published:** 2018-12-27

**Authors:** Tsutomu Wada, Azusa Sameshima, Rika Yonezawa, Mayuko Morita, Kanae Sawakawa, Hiroshi Tsuneki, Toshiyasu Sasaoka, Shigeru Saito

**Affiliations:** 1 Department of Clinical Pharmacology, University of Toyama, Toyama, Toyama, Japan; 2 Department of Obstetrics and Gynecology, University of Toyama, Toyama, Toyama, Japan; Radboud University Medical Centre, NETHERLANDS

## Abstract

Obesity and diabetes increase the risk of depression, and the incidence of these conditions increases rapidly after menopause, but few animal models of postmenopausal obesity have been available. We developed a mouse model of postmenopausal obesity that exhibited anxiety and depressive phenotypes in behavioral tests. To examine the effect of estradiol (E2) in the model, we prepared 4 experimental groups: 1) control, sham-operated female C57BL/6 mice fed a regular diet; 2) OVX-HF, ovariectomized (OVX) mice fed a high-fat diet (HF); 3) E2-SC, OVX-HF mice administered subcutaneous (SC) E2 (50 μg/kg/day); and 4) E2-ICV, OVX-HF mice administered intracerebroventricular (ICV) E2 (1 μg/kg/day). OVX-HF mice exhibited anxiety phenotypes in the open field test, but not in the light-dark box test, and E2 treatment via both routes effectively ameliorated it. OVX-HF mice demonstrated depressive phenotypes in the tail suspension test and forced swim test. Both E2 treatments achieved significant improvement in the tail suspension test, but not in the forced swim test. Serum corticosterone levels did not differ among the groups. Hippocampal expression of glucocorticoid receptor mRNA and serotonin 1A receptor mRNA was significantly increased in OVX-HF mice and was decreased in E2-treated mice. The hypothalamic level of pro-brain-derived neurotrophic factor (proBDNF) protein was tended to decrease in OVX-HF mice, but neither E2 treatment increased it. Since this mouse model exhibited anxiety and depressive phenotypes in relatively short experimental periods without genetic manipulations, it would be useful for further exploring psychiatric phenotypes or screening of therapeutic candidates in postmenopausal obesity.

## Introduction

When menopause occurs, the serum level of estrogen decreases rapidly in women around the age of 50 years, causing a variety of vasomotor and neuropsychiatric symptoms [1]. In particular, anxiety and depression are significantly associated with menopause and impairment of the quality of life. Several reports have indicated that women are more susceptible to developing anxiety disorders than men [2–4], especially after menopause [5]. The prevalence of depression is twice as high as in women as in men^4^ and the peak onset of depression is observed around the time of menopause [6–8], indicating that gonadal hormones contribute to protection against these emotional disorders.

Estrogen therapy is reported to be beneficial for alleviating menopause-related depressive symptoms [9]. However, the effectiveness of treatment varies among clinical studies [10, 11], with the route of estrogen administration (oral or transdermal) and the presence of obesity being suggested as possible factors that contribute to such discrepancies [12].

Obesity and diabetes are common risk factors for depression, and the incidence of both conditions increases markedly after menopause [13, 14]. Despite the importance of these factors in relation to emotional disorders, there has been no adequate postmenopausal mouse model of obesity for evaluating the efficacy of estrogen for amelioration of anxiety and depression. We have previously reported the mice model of postmenopausal obesity that exhibited metabolic characteristics of the condition such as obesity by decreased energy expenditure, glucose intolerance, and insulin resistance by performing OVX and feeding a high-fat diet (HF) [15]. In this study, we attempted to characterize psychological phenotypes of these mice to establish the mice model of postmenopausal obesity adequately reflecting features of both metabolic and psychological conditions. Since previous animal studies have demonstrated the beneficial effects of systemic estrogen treatment on anxiety and depression in a postmenopausal ovariectomized (OVX) mouse model [16–19], we further investigated the impact of brain versus systemic action of estrogen by comparison between the administration via subcutaneous (SC) and ICV route on anxiety and depressive phenotypes in the mice model.

## Materials and methods

### Animals and experimental design

**Fig 1:**
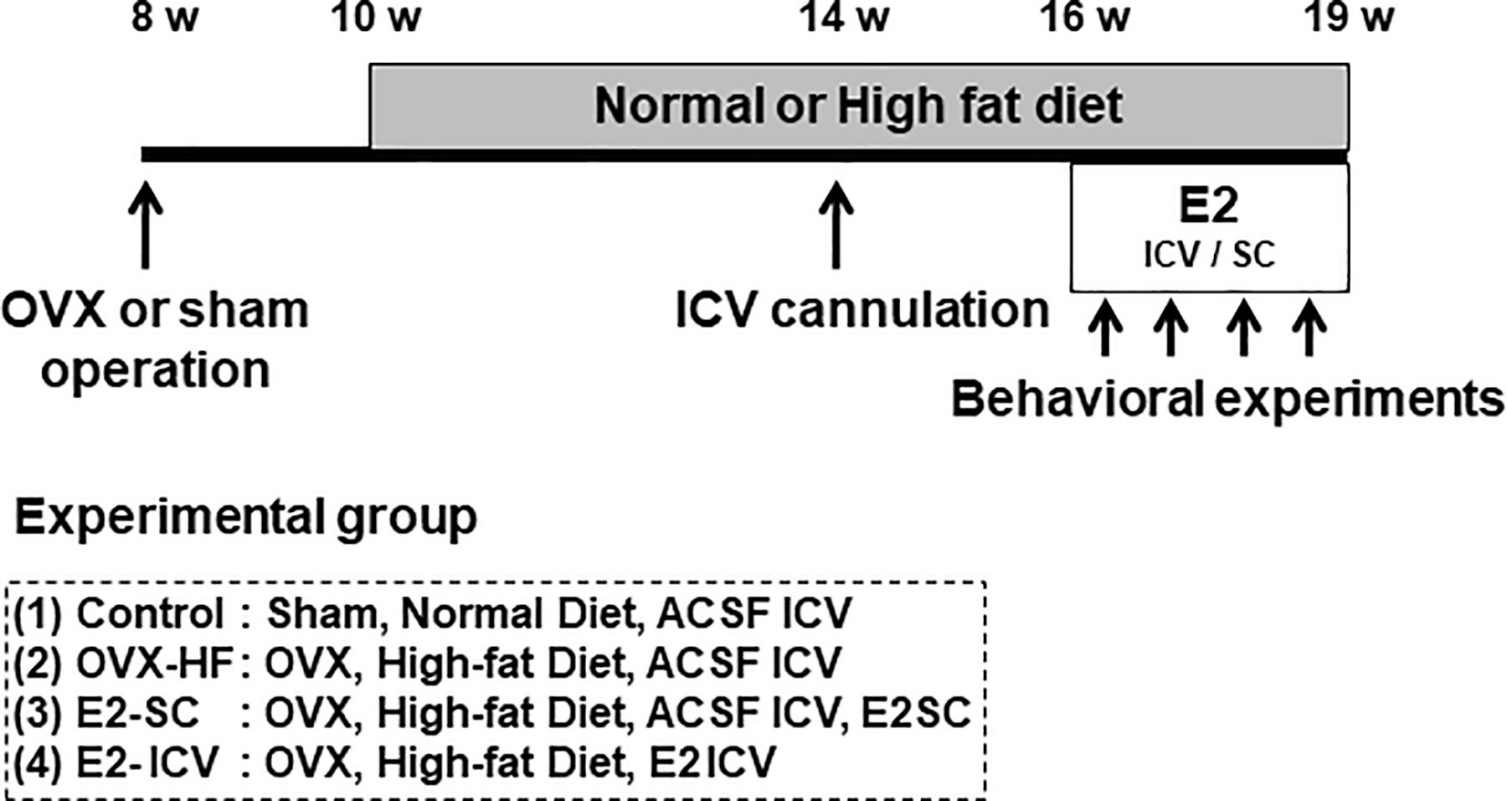
Research protocol. Eight-week-old female C57BL/6J mice maintained for 2 weeks after ovariectomy (OVX) or sham operation were fed a standard diet or a 60% high-fat diet for 4 weeks. At the age of 14 weeks, an intracerebroventriclar (ICV) cannula was implanted, and artificial cerebrospinal fluid (ACSF) was continuously infused into the lateral ventricle by an osmotic pump. At the age of 16 weeks, continuous subcutaneous administration of E2 (50 μg/kg/day) was conducted in the E2-SC group, or E2 (1 μg/kg/day) was infused via the ICV cannula in the E2-ICV group. Behavioral tests were conducted during 3 weeks of E2 administration.

Mice were maintained with standard lighting (12 h light-dark cycle), temperature (24 ± 1°C), and humidity (55 ± 10%), and were fed a normal diet containing 4.8% fat (CE-12; Clea Japan, Tokyo, Japan) and were provided with water *ad libitum*. All experimental procedures for this study were approved by the Animal Experiments Committee of the University of Toyama (Protocol number: A2011 PHA-17 and A2014 PHA-8). All surgery was performed under sodium pentobarbital anesthesia, and all efforts were made to minimize suffering. The experimental groups of mice are outlined in Fig 1. Eight-week-old female C57BL6/J mice purchased from Japan SLC (Shizuoka, Japan) either underwent OVX or sham operation. At the age of 10 weeks, the normal diet was changed to a high-fat diet (HF; 60% fat diet, D12492; Research Diets, New Brunswick, NJ). At the age of 14 weeks, an ICV cannula attached to an osmotic pump was implanted into the left lateral ventricle (0.3 mm posterior to the bregma, 0.9 mm lateral to the central sulcus, and 2.5 mm below the skull) of each mouse under anesthesia. Artificial cerebrospinal fluid (ACSF; Tocris Bioscience, Bristol, UK) was continuously injected by the osmotic pump (Alzet Brain Infusion Kits3, Alzet Osmotic pump model 1004; DURECT, Cupertino, CA), which was placed in a subcutaneous pocket on the back [20]. After a 2-week recovery period (16 weeks of age), the mice were divided into 4 groups. The control group was sham-operated mice fed normal diet and continuously administered ACSF via the ICV cannula. The OVX-HF group was OVX mice fed HF and continuously administered ACSF via the ICV cannula. The E2-SC group was OVX mice fed HF with subcutaneous administration of soluble E2 (Sigma-Aldrich, St. Louis, MO, E2 dose; 50 μg/kg/day) from another pump implanted on the other side of the back. The E2-ICV group was OVX mice fed HF with ICV administration of E2; the osmotic pump containing ACSF was replaced with a pump containing E2 (1 μg/kg/day) at 1/50 of the concentration for subcutaneous E2. Except for the E2-ICV group, the mice received ICV administration of ACSF via osmotic pump. The serum E2 level of the E2-SC group was similar to that in sham-operated mice, while delivery of E2 directly to the central nervous system in the E2-ICV group induced beneficial effects on metabolism without affecting the serum E2 concentration [15]. Approximately 3 to 5 mice were housed in the same cage before ICV cannulation, whereas each mouse was housed in the separate cage after cannulation to avoid desorption of cannulae. Behavioral experiments were conducted during E2 administration, and then the mice were sacrificed under anesthesia and analyzed (Fig 1).

### Behavioral experiments

Open field test: This test was conducted 3 or 4 days after initiation of E2 treatment and was performed for 25 min in an open field apparatus (H 30 cm × W 45 cm × L 45 cm). The floor of the apparatus was hypothetically divided into 25 squares (16 outer and 9 inner), and searching activity of the mice was recorded on video. The number of times each mouse crossed into the central area and the time spent in the central area were determined as indexes of the level of anxiety.

Light-dark box test: This test was conducted 7 or 8 days after initiation of E2 treatment. The test apparatus consisted of a black plastic box and a transparent plastic box (dark and light boxes, respectively; dimensions: 17 cm × 17 cm × 17 cm). A tunnel (H 4 cm × W 4 cm × L 10 cm) allowed the mice to move freely between the two boxes. The number of times mice traversed the tunnel and the time spent in the light box were determined during a 10-min period, and were utilized as indexes of general motor activity and the level of anxiety, respectively.

Tail suspension test: This test was conducted at 12 or 13 days after initiation of E2 treatment. After a small piece of adhesive tape was attached approximately 2 cm from the tip of the tail, each mouse was suspended for a period of 10 min from a hook 5 cm fixed to the ceiling of an open front box (H 22 cm × W 17 cm × L 15 cm). The mice made efforts to escape and then stopped moving after several attempts. The duration of immobility was recorded and was utilized as an index of depression-like behavior.

Forced swim test: This test was done at 20 or 21 days after initiation of E2 treatment. On day 1, each mouse was placed individually in a plastic cylinder (Φ 22 cm, H 25 cm) filled with water (maintained at 25°C) to a depth of 12 cm. The mice were forced to swim for 15 min and then returned to their cages. On day 2, mice were placed into the cylinder again and forced to swim for 6 min. The duration of immobility was recorded during the last 5 min of the session and was utilized as an index of depression-like behavior [21].

Numbers of mice used for each experiment are described in the figure legend.

### Real-time quantitative PCR

Extraction of RNA, reverse transcription, and real-time PCR using SYBR green were performed as described previously [15, 22, 23]. Expression of the target mRNAs was calculated as a ratio relative to that of S18 ribosomal protein. The following primer sequences were used for real-time PCR: glucocorticoid receptor primers, 5’-CTCTACCCTGCATGTATGACCA-3’ (forward) and 5’-TGGCTCTTCAGACCTTCCTTAG-3’ (reverse); 5-HT_1A_ primers, 5’-TCGCTCACTTGGCTCATTGGCTTT-3’ (forward) and 5’-TTCCACCTTCTTGACCGTCTTGCG-3’ (reverse); and S18 ribosomal protein primers, 5’- AGTTCCAGCACATTTTGCGAG-3’ (forward) and 5’- TCATCCTCCGTGAGTTCTCCA-3’ (reverse).

### Western blot analysis

The hippocampus of each mouse was dissected, frozen in liquid nitrogen, and stored at -80°C until use. Then the tissues were lysed and subjected to western blotting, as described previously [24]. In brief, the tissue lysates were separated by SDS-PAGE and transferred to membranes for immunoblotting with a primary antibody for brain-derived neurotrophic factor (BDNF) (Alomone Labs, Jerusalem, Israel). Then the membranes were subjected to direct densitometric analysis using an LAS-4000 lumino image analysis system (Fujifilm, Tokyo, Japan) [22, 25].

### Measurement of serum parameters

After mice were deprived of food overnight, blood samples were collected from the abdominal aorta under anesthesia and serum was separated by centrifugation at 15,000 g for 5 min. Serum corticosterone levels were measured with a corticosterone enzyme immunoassay kit (Cayman Chemical, Ann Arber, ML). The analyses were conducted in duplicate. The intra-assay coefficients of variation were less than 10% in each analysis.

### Statistical analysis

Results are displayed by using Turkey style box plots, with data points outside the lower and upper quartiles being indicated as circles. Results are expressed as the mean ± SE. Statistical significance was determined by the Mann-Whitney U-test after the two-tailed Kruskal Wallis H-test, and *P*<0.05 was considered significant.

## Results

### Basic characteristics of each experimental groups of mice

We initially characterized body and tissue weights in each group of mice (Table 1). Body weight was significantly higher in the OVX-HF group (*P*<0.01) compared with the control group. At 19 weeks old, both the body weight (*P*<0.01) and the perigonadal fat weight (*P*<0.01) were significantly decreased by either E2 treatment. Compared with the control mice, the uterine weight was significantly decreased in the OVX-HF group (*P*<0.01) and the E2-ICV group (*P*<0.01), but was slightly increased in the E2-SC group (*P*<0.01), possibly due to continuous exposure to E2 that was not dependent on the estrus cycle [15]. These results indicated that ICV administration of E2 had no direct peripheral effects under the present experimental conditions.

**Table 1:** Body weight and tissue weights of the mice.

	Control	OVX-HF	E2-SC	E2-ICV
8 weeks old (g)	17.2 ± 0.2	17.6 ± 0.2	17.5 ± 0.3	17.8 ± 0.3
16 weeks old (g)	21.7 ± 0.6	27.5 ± 0.8^**^	28.4 ± 1.0^**^	27.2 ± 0.5^**^
9 weeks old (g)	22.5 ± 0.2	29.9 ± 0.6^**^	26.4 ± 0.6^**^^††^	26.8 ± 0.8^**^^†^
Uterine (mg)	71.4 ± 9.9	9.7 ± 1.0^**^	116.7 ± 3.6^**^^††^	10.6 ± 0.9^**^^§§^
Perigonadal fat (mg)	0.14 ± 0.01	1.15 ± 0.09^**^	0.32 ± 0.07^*^^††^	0.80 ± 0.12^**^^†^^§§^

Body weights at 8 weeks old (before OVX), 16 weeks old (before E2 treatment), and 19 weeks old (after all behavioral experiments), and tissue weights at sacrifice (19 weeks old) in each group. Values are expressed as the mean ± SE (n = 10–11).

**P < 0.01 vs. the control group

^†^*P* < 0.05

^††^*P* < 0.01 vs. the OVX-HF group

^§§^*P* < 0.01, E2-SC group vs. E2-ICV group.

### Effect of central and peripheral E2 administration on anxiety phenotypes

**Fig 2:**
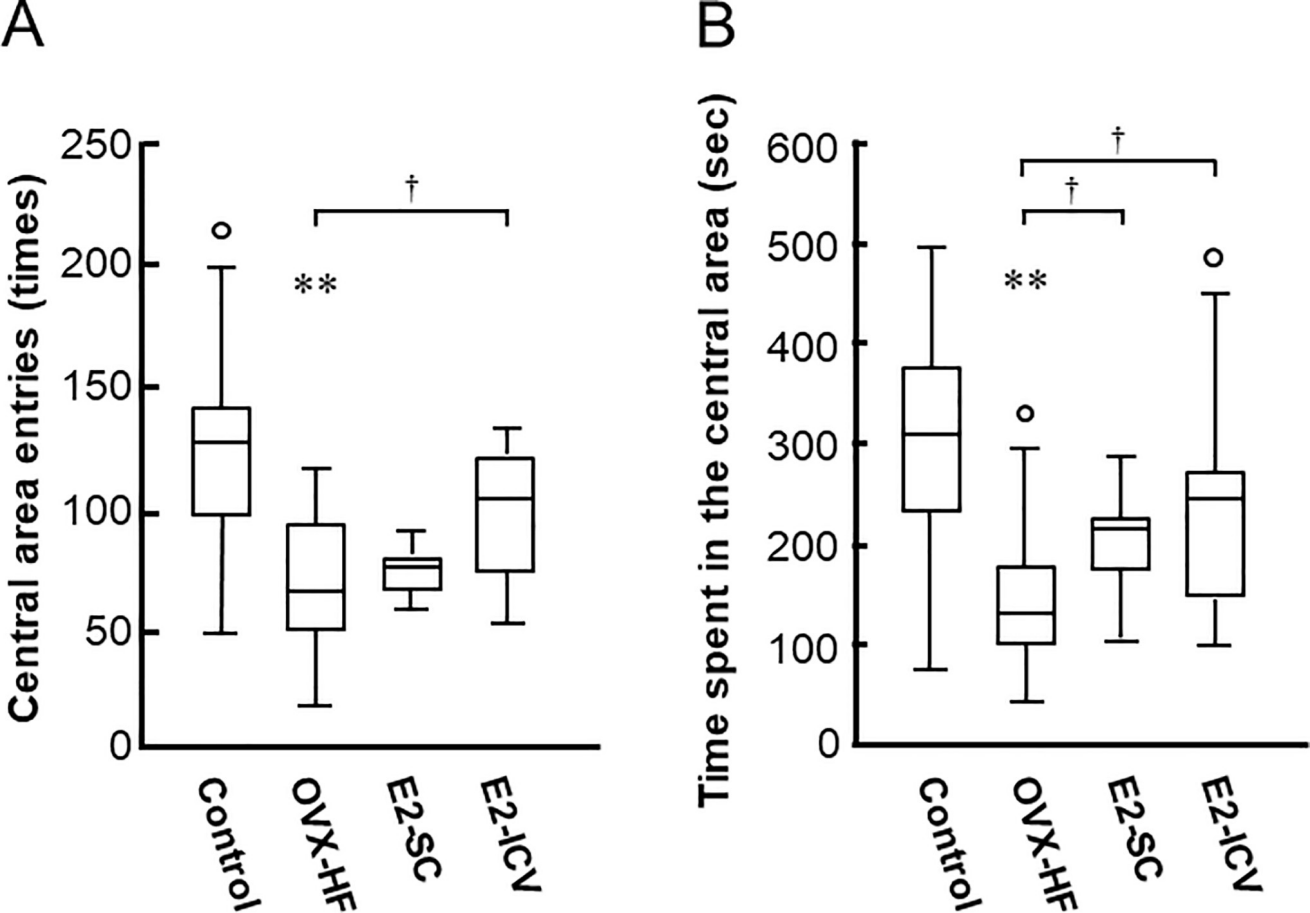
Effects of E2 administration on anxiety phenotypes in the open field test. Mice were placed at the center of an open field apparatus. The number of crosses into the central area (A) and the time spent in the central area (B) during a 25-min observation period were determined as indexes of the level of anxiety. Results are shown as Turkey style box plots with data falling outside the lower and upper quartiles plotted as circles. Values are the mean ± SE (control, n = 16; OVX-HF, n = 18; E2-SC, n = 11; E2-ICV, n = 10). *P* values were determined by the two-tailed Kruskal Wallis H-test with the Mann-Whitney U-test. **P<0.01 vs. the control group; ^**†**^*p*<0.05 vs. the OVX-HF group.

We investigated anxiety phenotypes in the mice by two typical behavioral tests. In the open field test, the number of crosses into the central area was significantly smaller and the time spent in the central area was significantly shorter in the OVX-HF group compared with the control group (*P*<0.01), indicating that OVX-HF mice exhibited phenotypic features of anxiety (Fig 2). Compared with the OVX-HF group, the number of crosses into the central area showed a significant increase in the E2-ICV group (*P*<0.05) and the time spent in the central area was significantly longer in both E2-treated groups (*P*<0.05).

**Fig 3:**
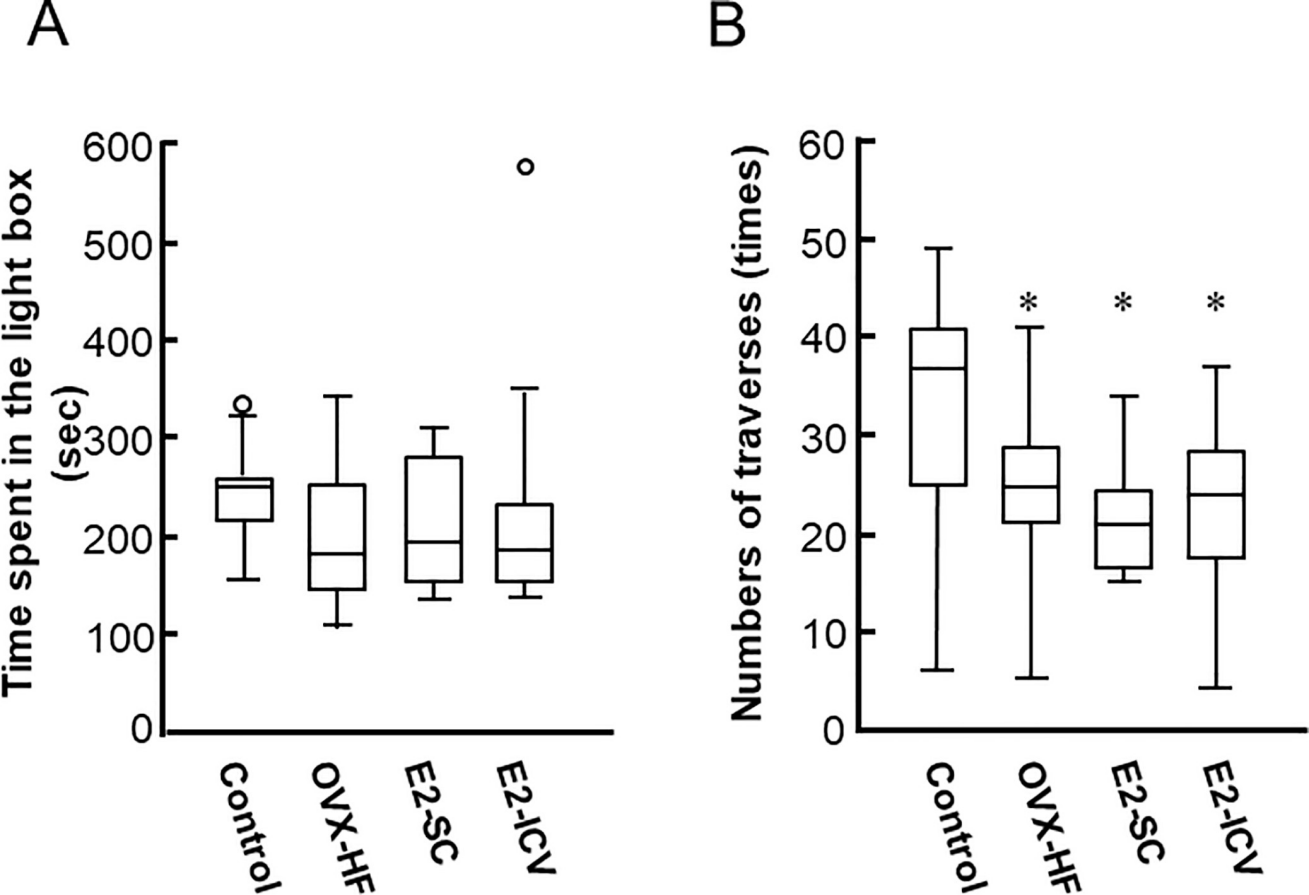
Effects of E2 administration on anxiety phenotypes in the light-dark box test. Mice were placed in the test apparatus consisting of a black plastic box (dark box) and a transparent plastic box (light box) connected by a small tunnel that allowed mice to move freely between the compartments. The number of traverses of the tunnel and the time spent in the light box during 10 min were analyzed (A). The time spent in the light box is considered to be an index of the level of anxiety and the number of tunnel traverses is an index of general motor activity (B). Results are shown as Turkey style box plots with data falling outside the lower and upper quartiles plotted as circles. Values are the mean ± SE (control, n = 13; OVX-HF, n = 17; E2-SC, n = 11; E2-ICV, n = 10). *P* values were determined by the two-tailed Kruskal Wallis H-test with the Mann-Whitney U-test. *P<0.05 vs. the control group; ^**†**^*p*<0.05 vs. the OVX-HF group.

In contrast, the time spent in the light box showed no difference among the 4 groups of mice in the light-dark box test (Fig 3A). The number of tunnel traverses, an index of general motor activity, was significantly decreased in the OVX-HF, E2-SC, and E2-ICV groups compared with the control group (P<0.05), and neither method of E2 administration improved this index compared with the OVX-HF group (Fig 3B). Taken together, these results indicated that the open field test may be more appropriate for evaluating anxiety in OVX-HF mice, because these mice with postmenopausal obesity exhibited anxiety phenotypes that were improved by estrogen administration.

### Effect of central and peripheral E2 administration on depression phenotypes

**Fig 4:**
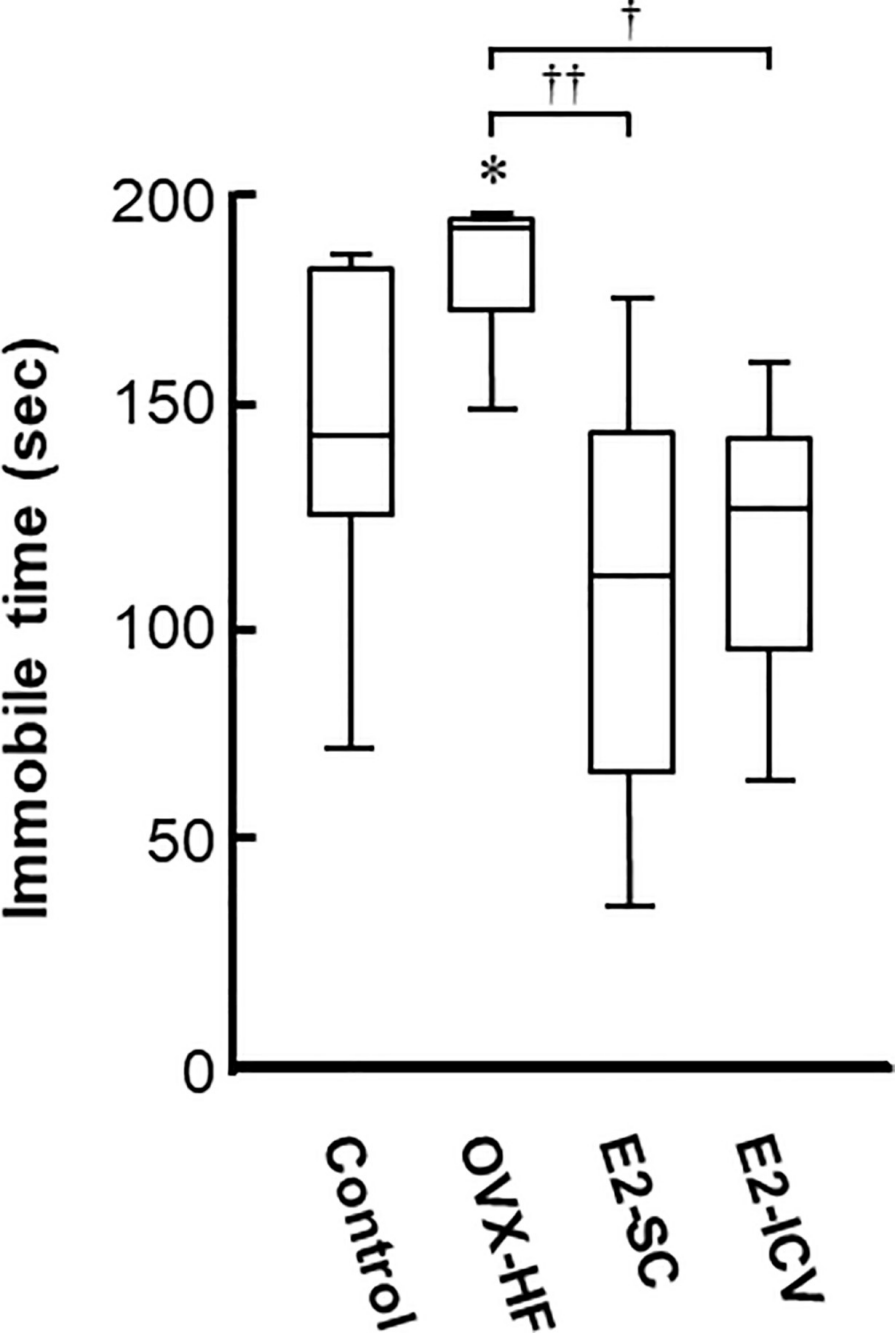
Effects of E2 administration on depression phenotypes in the tail suspension test. Mice were taken from the home cage and a small piece of adhesive tape was attached approximately 2 cm from tip of the tail. Then mice were individually suspended on a hook 5 cm from ceiling in the open box for a period of 10 min. After the mice stopped making efforts to escape after several attempts, the duration of immobility was recorded as an index of depression-like behavior. Results are shown as Turkey style box plots. Values are the mean ± SE (control, n = 9; OVX-HF, n = 7; E2-SC, n = 11; E2-ICV, n = 8). *P* values were determined by the two-tailed Kruskal Wallis H-test with the Mann-Whitney U-test. *P<0.05 vs. the control group; ^**††**^*p*<0.01, ^**†**^*p*<0.05 vs. the OVX-HF group.

We next examined depression phenotypes in these mice by two behavioral tests. The tail suspension test showed that the immobility time was significantly longer in OVX-HF mice compared with control mice (*P*<0.05). In addition, the immobility time was significantly reduced in both E2-treated groups compared with OVX-HF mice (E2-SC group: *P*<0.01, E2-ICV group: *P*<0.05) (Fig 4).

**Fig 5:**
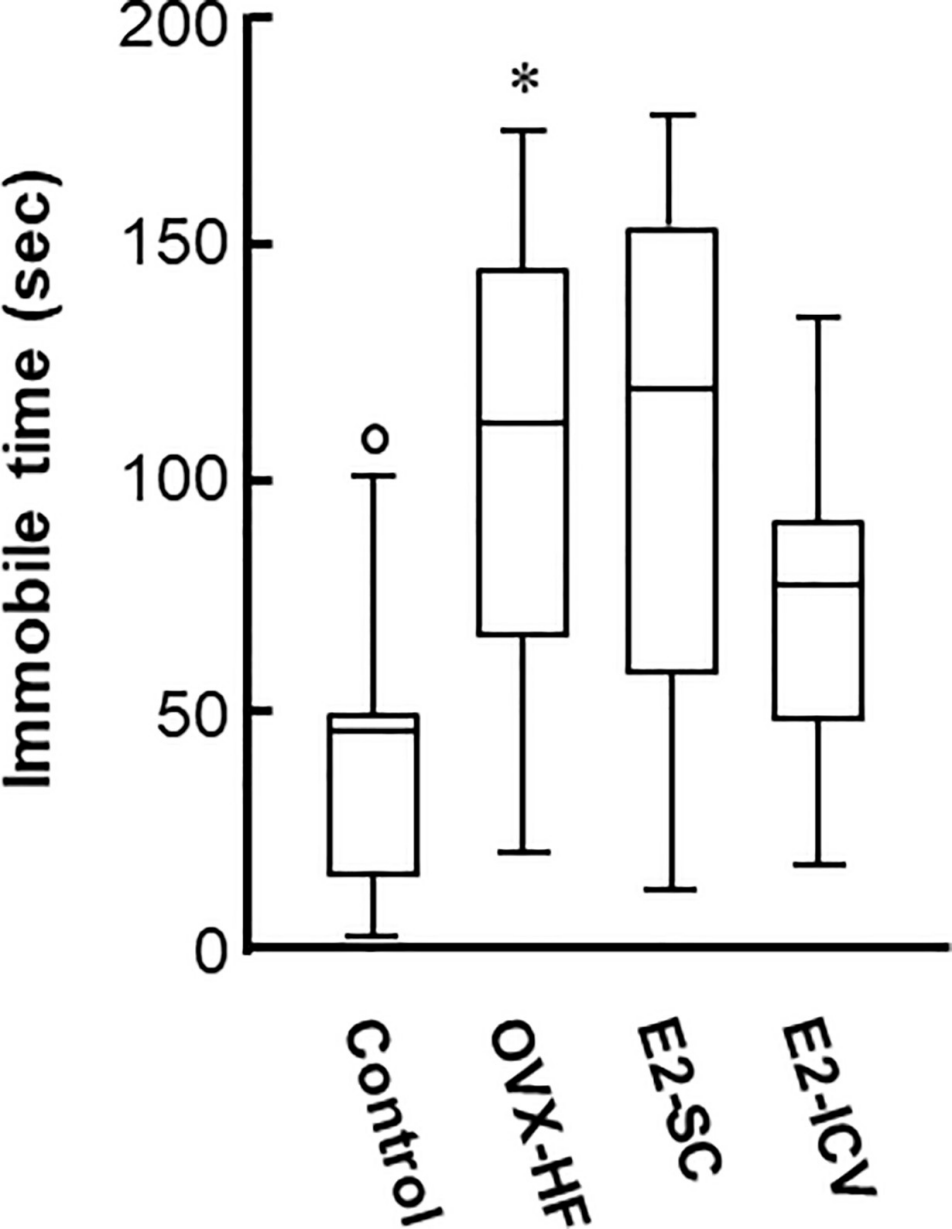
Effects of E2 administration on depression phenotypes in the forced swim test. On day 1, mice were placed in a plastic cylinder filled with water, forced to swim for 15 min, and then returned to the home cage. On day 2, mice were placed into the water again and forced to swim for 6 min. The duration of immobility was recorded during the last 5 min of the session as an index of depression-like behavior. Results are shown as Turkey style box plots with data falling outside the lower and upper quartiles plotted as circles. Values are the mean ± SE (control, n = 8; OVX-HF, n = 9; E2-SC, n = 11; E2-ICV, n = 8). *P* values were determined by the two-tailed Kruskal Wallis H-test with the Mann-Whitney U-test. **P<0.01, *P<0.05 vs. the control group; ^**††**^*p*<0.01 vs. the OVX-HF group.

In the forced swim test, the immobility time was also significantly longer in OVX-HF mice compared with control mice (*P*<0.01). Although the immobility time was decreased in the E2-ICV group, it did not show a significant difference in either E2-treated group (Fig 5). The results of these two behavioral tests indicated that OVX-HFD mice with postmenopausal obesity exhibit depression phenotypes, and that E2 partially reverses these phenotypes.

### Mechanism underlying E2-mediated amelioration of anxiety and depression

**Fig 6:**
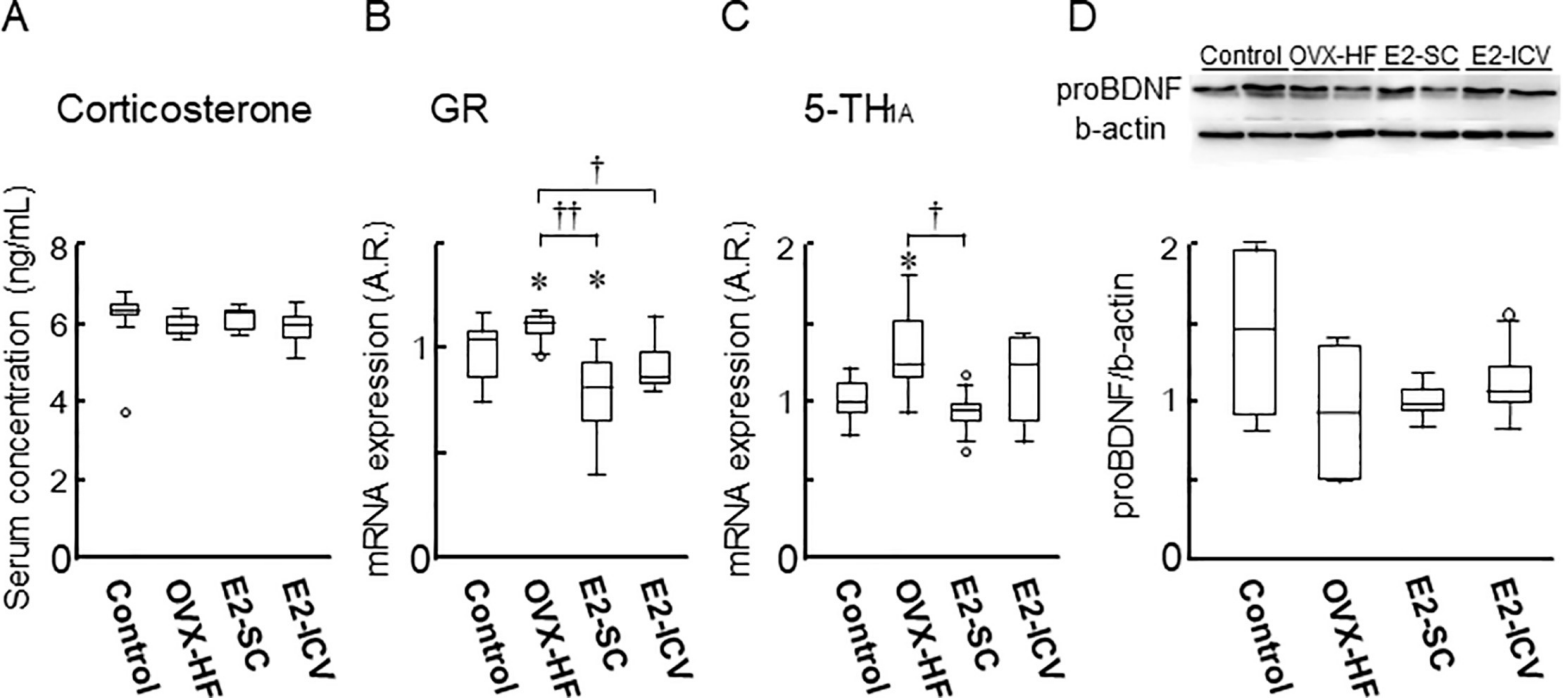
Biological analysis of the mice after behavioral experiments. Mice were sacrificed and samples were obtained after conducting all of the behavioral experiments. The serum corticosterone level was determined with an ELISA kit (A). Expression of GR mRNA (B) and 5-TH_1A_ mRNA (C) in the hippocampus was determined by real-time PCR. The level of proBDNF protein in the hippocampus was analyzed by western blotting (D). Values are the mean ± SE (n = 7-11/group). *P* values were determined by the two-tailed Kruskal Wallis H-test with the Mann-Whitney U-test. *P<0.05 vs. the control group; ^**††**^*p*<0.01 vs. the OVX-HF group.

To investigate the mechanism by which E2 improved anxiety and depression phenotypes in these mice, we examined serum corticosterone levels, but found no differences among the 4 groups (Fig 6A). However, hippocampal expression of glucocorticoid receptor (GR) mRNA was increased in OVX-HF mice compared with control mice (*P*<0.05), while its expression was decreased in the E2-SC group (*P*<0.01) and the E2-ICV group (*P*<0.05) compared with the OVX-HF group (Fig 6B). In addition, hippocampal expression of mRNA for serotonin receptor 1A (5-HT_1A_), a major serotonin receptor subtype associated with anxiety and depression, was significantly increased in OVX-HF mice compared with control mice (*P*<0.05). Expression of 5-HT_1A_ mRNA was significantly decreased in the E2-SC group (*P*<0.05) compared with the OVX-HF group, but not in the E2-ICV group (Fig 6C). On the other hand, the hippocampal content of pro-brain derived neurotrophic factor (BDNF) protein did not show any significant differences among the groups, although it was lower in OVX-HF mice (Fig 6D).

## Discussion

Women are more likely to suffer from mood disorders associated with hormonal fluctuations in the premenstrual, postpartum, and perimenopausal periods. From 15 to 50% of perimenopausal women experience depressive symptoms [10], which may impair their quality of life, and the effectiveness of hormone replacement therapy for mood disorders in perimenopausal women has been reported [26, 27]. Estrogen therapy is currently utilized for these psychological disorders in perimenopausal women [11, 28], but appears to be less effective when used for postmenopausal depression [29]. The prevalence of obesity and diabetes is known to increase in menopausal women [12], and these metabolic diseases are risk factors for psychiatric disorders. Despite the potential clinical importance, there has been no mouse model of postmenopausal obesity that is adequate for investigation of anxiety and depression in these women. We developed the OVX-HF mouse as a novel model of postmenopausal obesity. We have previously shown that these mice develop obesity with abnormal glucose, lipid, and energy metabolism [15]. In the present study, we demonstrated that these mice also show typical phenotypes of postmenopausal emotional disorders (including anxiety and depression) in behavioral tests. Based on these findings, our mouse model appears to be useful for investigation of emotional disorders associated with postmenopausal obesity.

Estrogen is the cardinal sex hormone in women that regulates sexual characteristics, the menstrual cycle, and glucose and energy homeostasis [30–32]. We have previously clarified the central and peripheral actions of E2 in our mouse model [15]. Central actions of E2 include promotion of lipolysis in white adipose tissue and thermogenesis in brown adipose tissue, suppression of gluconeogenesis in the liver, and elevation of spontaneous physical activity and energy expenditure, all of which contributes to reduction of the body fat mass and enhancement of insulin sensitivity. Peripherally, estrogen directly counters adiposity and improves insulin sensitivity by suppressing the expression of lipogenic genes and chronic inflammation in adipose tissue. These central and peripheral actions of estrogen appear to coordinately improve systemic glucose homeostasis in this mouse model.

In the present study, we investigated the effectiveness of E2 administration for phenotypic features of anxiety and depression in our mouse model. We found that ICV administration of E2 increased the abnormally low number of crossings into the central area and prolonged the time spent in the central area in the open field test, as well as reducing the immobile time in the tail suspension test (Figs 2 and 4). Subcutaneous administration of E2 also prolonged the time spent in the central area in the open field test and reduced the immobile time in the tail suspension test (Figs 2 and 4). These results strongly suggest that E2 is beneficial for anxiety and depression in this mouse model of menopausal obesity, regardless of the administration route. A possible mechanism of obesity-associated depressive behaviors is chronic inflammation in the brain [33, 34]. Since estrogen has an anti-inflammation activity, especially in myeloid cells [35, 36], one can assume that estrogen replacement improves the chronic inflammation in the brain of OVX-HF mice. However, mRNA expressions of TNFα and IL6 per se were not significantly increased in the hippocampus of OVX-HF mice, and estrogen treatment did not affect the expression (S1 Fig). Since chronic inflammations in the visceral adipose tissue were significantly attenuated by estrogen treatment in the same animal model [15], estrogen induced reduction of circulating proinflammatory cytokines may be partly involved in the amelioration of behavioral phenotypes in the current model.

The hypothalamic-pituitary-adrenal (HPA) axis is activated by stress and contributes to elevation of the serum corticosterone level in anxiety and depression [37], and impairment of glucocorticoid receptor (GR) function has been demonstrated in patients with depressive disorders [38]. Therefore, maintenance of the HPA system is expected to be a target for treating depression [38, 39]. Indeed, both forebrain-specific GR deletion and GR overexpression lead to anxiety and depression phenotypes in mice [40, 41]. In the present study, the serum corticosterone level showed no significant differences among the four groups of mice (Fig 6A), whereas E2 treatment by either route ameliorated the decrease of hippocampal GR expression in OVX-HF mice (Fig 6B). In this regard, anxiety phenotypes caused by central administration of a GR agonist is ameliorated by systemic treatment with an estrogen receptor β agonist in rats [42]. To explore the possible role of the estrogen receptor in E2-mediated amelioration of anxiety and depressive phenotypes in our mouse model, we examined its expression in the hippocampus, but we found that the levels of mRNA for estrogen receptor α, estrogen receptor β, and a G protein-coupled receptor (GPR30) were indistinguishable among the 4 groups of mice (S2 Fig). Accordingly, further studies will be needed to clarify the link between sex steroid hormones and their receptors in the development and E2-mediated amelioration of emotional disorders.

Serotoninergic systems have various neuronal functions [43]. Selective serotonin reuptake inhibitors (SSRI) and serotonin and norepinephrine reuptake inhibitors (SNRI) are widely utilized for the treatment of depression and anxiety disorders by reinforcing serotonin or norepinephrine systems, respectively [44, 45]. Estrogen has been reported to strengthen serotonin signaling by acting on postsynaptic serotonin receptors, increasing serotonin receptor expression, and altering serotonin transporter gene expression [46–49]. In addition, estrogen has been shown to increase serotonin levels by suppressing monoamine oxidase and catechol-O-methyltransferase activity [50, 51]. It is also known that serotonin modulates BDNF synthesis through the phosphorylation of cAMP response element binding protein (CREB) [52–54]. Therefore, it can be assumed that the decline of estrogen in menopause could have adverse neuropsychiatric effects. 5-HT_1A_ is one of the major 5-HT receptors in the brain, and is known to be associated with memory, anxiety, and depression [55–57]. It is a Gi-coupled receptor that negatively regulates CREB-mediated BDNF expression [58, 59]. Accordingly, we examined the expression of 5-HT_1A_ and BDNF in our mouse model. We found elevated expression of 5-HT_1A_ mRNA along with a slight decrease of proBDNF protein in the hippocampus of OVX-HF mice. In addition, the increase of 5-TH_1A_ expression in OVX-HF mice was significantly attenuated in E2-SC mice. However, E2 treatment by either route did not restore the reduced proBDNF level under our experimental conditions (Fig 6). This led us to conclude that the 5-TH_1A_/CREB/BDNF pathway is not strongly involved in the mechanism by which estrogen improves anxiety and depression in this mouse model. In this context, estradiol replacement has been shown to ameliorate anxiety and depressive behavior along with restoration of hypothalamic BDNF levels in ovariectomized female rats [60]. Notably, it was reported that a high-fat diet reduces BDNF expression in the hippocampus [61], suggesting that the impact of obesity may block the beneficial influence of estrogen on restoration of BDNF. Because the precise reason why estrogen did not affect BDNF levels in the current study is unclear, further investigation will be needed to clarify the mechanism by which the central actions of estrogen improve emotional behavior in mice with menopausal obesity.

Although our model system showed the anxiety and depressive phenotypes in behavioral tests, several limitations can be assumed. First, the age of mice is younger than really-menopaused mouse, and the mice do not reflect whole pathogenic condition of postmenopausal obesity. Along this line, some biological parameter did not represent clinical aspect of depression or anxiety; *i*.*g*. serum corticosterone level did not elevate in the OVX-HF mice (Fig 6A). Second, the anxiety phenotype was observed in the open field test, but not in the light-dark box test. Third, the difference in behavioral abnormality between OVX mice fed chow diet and high-fat diet (HF) was not compared in the current study. Since anxiety and depression phenotypes have been reported in either OVX mice or HF-fed mice [16–19, 33, 34, 62], the current study focused on the combination of menopause and HF-feeding in the development of behavioral abnormalities. Despite these limitations, the mice possess crucial beneficial points for employment as the model. In this regard, the model needs relatively shorter times for the preparation, and it did not require genetic manipulations.

Interestingly, the effects of central E2 administration on emotional disorders were similar to those of systemic administration; although the dosage of central E2 was only 1/50 of systemic administration. We have previously shown a prominent beneficial impact of central E2 administration on glucose and energy metabolism in our mouse model of postmenopausal obesity. Taken together, our findings suggest the usefulness of a novel method of E2 replacement, namely centrally targeted E2 treatment. By using the current system, we would like to study whether estrogen improves obesity-associated depression or anxiety phenotypes in premenopause young animals in our future project, since estrogen has been shown to possess pleiotropic effects on neural functions such as increase of BDNF and attenuation of chronic inflammation. In addition, we encourage development of a trans-nasal estrogen administration system that would be beneficial for treatment of psychiatric disorders as well as metabolic diseases (including obesity and diabetes) associated with menopause, without concern about peripheral complications such as the development of E2-dependent cancer and venous thromboembolism.

In conclusion, we reported a mouse model of postmenopausal obesity that exhibited anxiety disorder and depression phenotypes, and these psychiatric phenotypes were improved by E2 replacement. The study with actually menopaused mice requires long experimental periods, and the experiment with genetically modified mice is subjected to regulation on use. Since a current mouse model did not use transgenic mice and exhibited anxiety and depressive phenotypes in relatively short time periods, it would be useful for further exploring psychiatric phenotypes or screening of therapeutic candidates in postmenopausal obesity.

## Supporting information

S1 Fig Expressions of proinflammatory cytokine in the hippocampus. Mice were sacrificed and samples were obtained after conducting all of the behavioral experiments. Expression of tumor necrosis factor α (TNFα) (A) and interleukin 6 (IL6) mRNA (B) in the hippocampus was determined by real-time PCR. The following primer sequences were used for real-time PCR: TNFα primers, 5’-AAGCCTGTAGCCCACGTCGTA-3’ (forward) and 5’-GGCACCACTAGTTGGTTGTCTTTG-3’ (reverse); IL6 primers, 5’-ATGGATGCTACCAAACTGGAT-3’ (forward) and 5’-TGAAGGACTCTGGCTTTGTCT-3’ (reverse). Results are shown as Turkey style box plots with data falling outside the lower and upper quartiles plotted as circles. Values are the mean ± SE (n = 6-11/group). *P* values were determined by the two-tailed Kruskal Wallis H-test with the Mann-Whitney U-test.
(TIF)

S2 Fig Expressions of estrogen receptors in the hippocampus. Mice were sacrificed and samples were obtained after conducting all of the behavioral experiments. Expression of estrogen receptor α (ERα) (A), estrogen receptor β (ERβ), and GPR30 mRNA (C) in the hippocampus was determined by real-time PCR. Results are shown as Turkey style box plots with data falling outside the lower and upper quartiles plotted as circles. The following primer sequences were used for real-time PCR: ERα primers, 5’-CCGTGTGCAATGACTATGCC-3’ (forward) and 5’-GTGCTTCAACATTCTCCCTCCTC-3’ (reverse); ERβ primers, 5’-ATGTGCTATGGCCAACTTC-3’ (forward) and 5’-TGGCGCTTGGACTAGTAAC-3’ (reverse); GPR30 primers, 5’-GATCGTTAGATTAACAGAGCAG-3’ (forward) and 5’-CCTGGGAGCCTGTTAGTCTCAG-3’ (reverse). Values are the mean ± SE (n = 6-11/group). *P* values were determined by the two-tailed Kruskal Wallis H-test with the Mann-Whitney U-test.
(TIF)
