# Supplementary figures and images for: Impact of central and peripheral estrogen treatment on anxiety and depression phenotypes in a mouse model of postmenopausal obesity
Source: PLoS One. 2018 Dec 27;13(12):e0209859. doi: 10.1371/journal.pone.0209859 (PMC6307752; doi:10.1371/journal.pone.0209859)

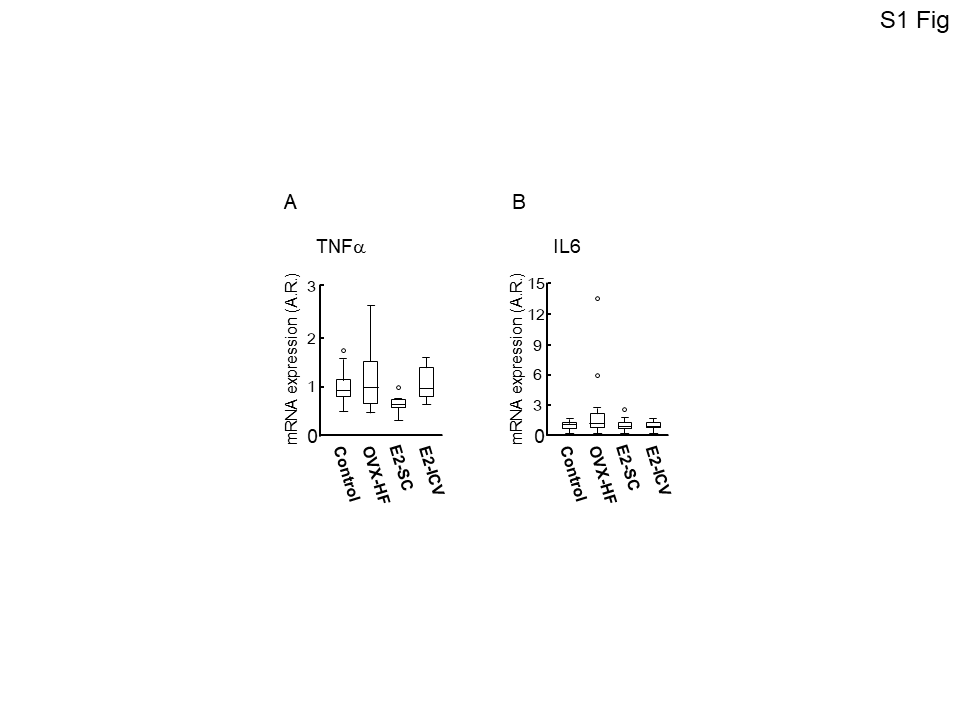

Supplement: S1 Fig — Mice were sacrificed and samples were obtained after conducting all of the behavioral experiments. Expression of tumor necrosis factor α (TNFα) (A) and interleukin 6 (IL6) mRNA (B) in the hippocampus was determined by real-time PCR. The following primer sequences were used for real-time PCR: TNFα primers, 5’-AAGCCTGTAGCCCACGTCGTA-3’ (forward) and 5’-GGCACCACTAGTTGGTTGTCTTTG-3’ (reverse); IL6 primers, 5’-ATGGATGCTACCAAACTGGAT-3’ (forward) and 5’-TGAAGGACTCTGGCTTTGTCT-3’ (reverse). Results are shown as Turkey style box plots with data falling outside the lower and upper quartiles plotted as circles. Values are the mean ± SE (n = 6-11/group). P values were determined by the two-tailed Kruskal Wallis H-test with the Mann-Whitney U-test. (TIF) [file pone.0209859.s001.tif]

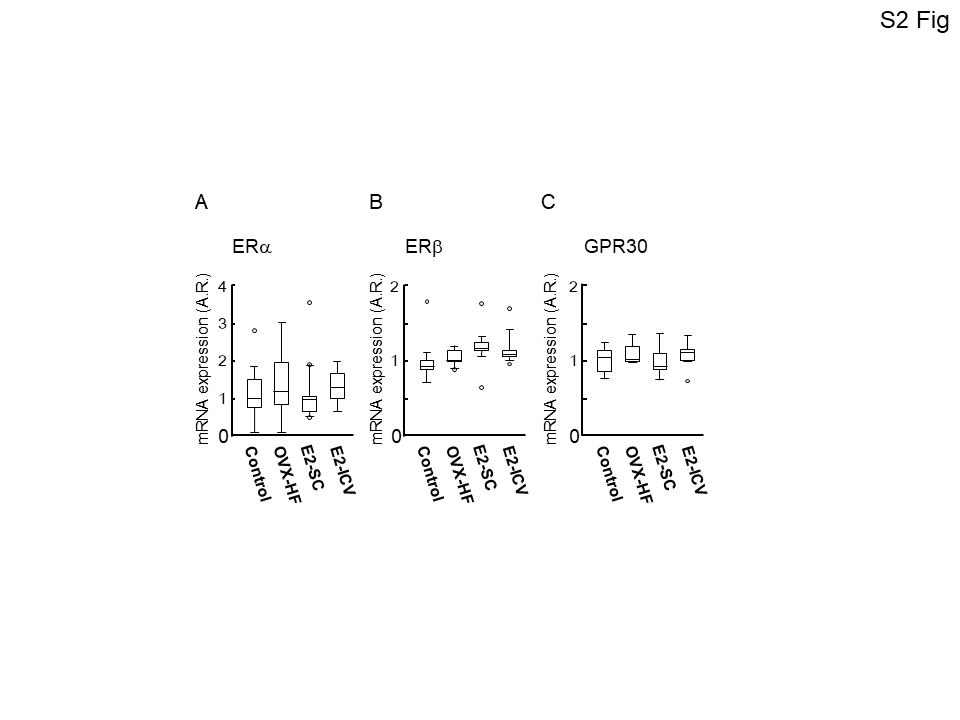

Supplement: S2 Fig — Mice were sacrificed and samples were obtained after conducting all of the behavioral experiments. Expression of estrogen receptor α (ERα) (A), estrogen receptor β (ERβ), and GPR30 mRNA (C) in the hippocampus was determined by real-time PCR. Results are shown as Turkey style box plots with data falling outside the lower and upper quartiles plotted as circles. The following primer sequences were used for real-time PCR: ERα primers, 5’-CCGTGTGCAATGACTATGCC-3’ (forward) and 5’-GTGCTTCAACATTCTCCCTCCTC-3’ (reverse); ERβ primers, 5’-ATGTGCTATGGCCAACTTC-3’ (forward) and 5’-TGGCGCTTGGACTAGTAAC-3’ (reverse); GPR30 primers, 5’-GATCGTTAGATTAACAGAGCAG-3’ (forward) and 5’-CCTGGGAGCCTGTTAGTCTCAG-3’ (reverse). Values are the mean ± SE (n = 6-11/group). P values were determined by the two-tailed Kruskal Wallis H-test with the Mann-Whitney U-test. (TIF) [file pone.0209859.s002.tif]
